# Mental health solutions for domestic violence victims amid COVID-19: a review of the literature

**DOI:** 10.1186/s12992-021-00710-7

**Published:** 2021-06-28

**Authors:** Zhaohui Su, Dean McDonnell, Stephanie Roth, Quanlei Li, Sabina Šegalo, Feng Shi, Shelly Wagers

**Affiliations:** 1 Center on Smart and Connected Health Technologies, Mays Cancer Center, School of Nursing, UT Health San Antonio, San Antonio, TX 78229 USA; 2 Department of Humanities, Institute of Technology Carlow, Carlow, R93 V960 Ireland; 3 Simmy and Harry Ginsburg Library, Temple University, Philadelphia, PA 19140 USA; 4 School of Nursing, Johns Hopkins University, Baltimore, MD 21205 USA; 5 Department of Microbiology, Faculty of Medicine, University of Sarajevo, 71000 Sarajevo, Bosnia and Herzegovina; 6 Department of Research and Development, Shanghai United Imaging Intelligence, 200232 Shanghai, China; 7 Department of Criminology, University of South Florida St. Petersburg, St. Petersburg, FL 33701 USA

**Keywords:** COVID-19, Coronavirus, SARS-CoV-2, Mental health, Domestic violence and abuse, Intimate partner violence, Interventions, Violence against women, Pandemic

## Abstract

**Background:**

Due to COVID-19, domestic violence victims face a range of mental health challenges, possibly resulting in substantial human and economic consequences. However, there is a lack of mental health interventions tailored to domestic violence victims and in the context of COVID-19. In this study, we aim to identify interventions that can improve domestic violence victims’ mental health amid the COVID-19 pandemic to address the research gap.

**Main text:**

Drawing insights from established COVID-19 review frameworks and a comprehensive review of PubMed literature, we obtained information on interventions that can address domestic violence victims’ mental health challenges amid COVID-19. We identified practical and timely solutions that can be utilized to address mental health challenges domestic violence victims face amid COVID-19, mainly focusing on (1) decreasing victims’ exposure to the abuser and (2) increasing victims’ access to mental health services.

**Conclusion:**

Domestic violence is a public health crisis that affects all demographics and could result in significant morbidity and mortality. In addition to emphasizing mental health challenges faced by domestic violence victims, multidisciplinary interventions are identified that could provide timely and practical solutions to domestic violence victims amid the pandemic, which range from tailored shelter home strategies, education programs, escape plans, laws and regulations, as well as more technology-based mental health solutions. There is a significant need for more multipronged and multidisciplinary strategies to address domestic violence amid and beyond the pandemic, particularly interventions that could capitalize on the ubiquity and cost-effectiveness of technology-based solutions.

## Background

Domestic violence is a global health crisis [1], with some statistics showing that one in every three individuals worldwide will experience domestic violence in some form [2]. Understood as a behavior focused on the oppression of another individual, causing significant hurt and trauma through physical, sexual, and mental harm [3], domestic violence is prevalent across all ages, ethnicities, and economic classes [4]. Amid the COVID-19 pandemic, domestic violence cases have grown exponentially worldwide [5–7], especially violence against women. Although lockdown has contributed to a 40% reduction in crime in Australia, there is a 5% rise in domestic violence cases [8–10]. Insights from Google Trends further support this finding, showing a 75% increase in online searches for domestic violence victims’ support [9].

Other countries, such as China, have also witnessed a dramatic rise in domestic violence cases [11]. Police reports from a city in Hubei Province, Jianli city, recorded a three-fold surge of violence against women cases, 90% influenced by isolation and lockdown due to COVID-19 [12]. Alarmingly, other statistics are increasing, too; across the United Kingdom (U.K.), police reports show that twice as many women were murdered by their domestic partners between March 23rd and April 12th, 2020 when compared against the average annual rates for the past decade [13]. It is important to note that while previous research has shown behavioral differences in the violence of men towards women and women towards men [14], recent papers concerning domestic violence show increasing frequency for both scenarios [15]. Take violence against men for instance. Research indicates that the prevalence of violence against men could range from 3.4 to 20.3% [16], an alarming trend that fuels the call for more research to untangle the cultural and societal perceptions of victimization [17, 18], especially factoring in the challenges associated with help-seeking behavior across victim demographics [19, 20].

Situations are even worst for violence against women—systematic research suggests that cases of domestic violence against women are often unreported or underreported [21–23]. In a study on domestic violence against women in Bosnia and Herzegovina, researchers found that only 5% of current domestic violence victims report their abuse to the police [24]. These sobering statistics underscore the soceital imperative for timely strategies and practical solutions to address the unprecedented challenges domestic violence victims face amid the pandemic. As a large body of research during COVID-19 focuses on violence faced by women, the narrative of this paper is in alignment with this. However, the majority of findings made throughout should be viewed as addressing domestic violence as a whole and aims to highlight the broad health consequences, particularly mental health consequences, of domestic violence against women during even beyond the COVID-19 pandemic.

The act and prevalence of violence against women, inevitably, can result in severe health consequences that often manifest in short-term and /or long-term physical and mental health issues [25, 26]. Compared with physical health consequences, mental illnesses might be less visible or tangible to the victims themselves and society. Mental health issues can manifest in a range of symptoms or as ‘asymptomatic’ in individuals who appear to be mentally healthy; and those can face severe and substantial mental health challenges that may go unnoticed and therefore left untreated [27]. The complex and multifaceted nature of mental health issues can often delay them from seeking help, which could further deteriorate their well-being and welfare [28]. Overall, mounting evidence suggests that domestic violence victims often face considerable mental health challenges [29–31].

Mental health is “a state of well-being in which every individual realizes his or her own potential, can cope with the normal stresses of life, can work productively and fruitfully, and is able to make a contribution to her or his community” [32]. Some research has shown that individuals seeking help from domestic violence support services report that 75% of these individuals have clinical posttraumatic stress symptoms, with depression and anxiety cases even more prevalent [30]. Similar to their western counterparts, domestic violence victims in China often face an array of mental health issues, including substance abuse, stress, anxiety, depression, and suicide [33–35]. In a study of 2987 domestic violence victims in China, researchers found that the prevalence of depression ranges from 65.8 to 75.8% across groups [36].

Overall, there is a consensus among researchers regarding the indispensable role of mental health in shaping an individuals’ overall health and well-being. The deterioration of mental health can affect both the psychological and physical aspects of human health [37–40]. Health experts have long warned that, due to its prevalence and severity, society is facing an epidemic of mental illness [41]. Amid a pandemic, which has already exerted significant human and economic consequences on public health [42–45], more ways to address domestic violence victims’ mental health challenges are needed [46–48].

Health interventions could be understood as tailored strategies or programs that aim to produce timely and cost-effective health solutions to a target audience [49, 50]. The need and benefits of tailored interventions have been well-documented in the literature [51–55]. Mounting research shows that mental health interventions can mitigate adverse effects caused by mental health issues and safeguard domestic violence victims’ overall health and well-being [46–48]. However, while health interventions have great potential, there is currently an alarming lack of mental health interventions tailored to domestic violence victims, particularly in the COVID-19 context [8–10]. Due to lockdown and social distancing measures, many mental health services and domestic violence support mechanisms, have either postponed or cancelled their services [8–10]; further compounding the lack of mental health solutions, a situation that many domestic violence victims face [56]. Therefore, to address the research gap, this paper aims to review the literature and identify existing interventions that can be adapted to the COVID-19 context to safeguard and improve domestic violence victims’ mental health and well-being.

## Main text

### Methods

Drawing insights from established COVID-19 review frameworks (e.g., [57]), and a comprehensive review of PubMed literature, we obtained information on interventions that can address domestic violence victims’ mental health challenges amid COVID-19. Furthermore, to ensure up-to-date evidence was obtained, verified news articles were also included in the review. Search terms used were: (“domestic violence” OR “intimate partner violence” OR “family violence” OR “spousal violence” OR abuse* OR batter* OR violen*) AND (intervention* OR trial* OR “randomized controlled trial*” OR RCT) AND (“COVID 19″ OR COVID-19 OR “coronavirus 2019″ OR “SAS-CoV-2″) AND (“mental health” OR “psychological health” OR “tele-health”).

#### Inclusion and exclusion criteria

Our inclusion criteria are listed in Table 1. Articles were excluded if (1) the study sample did not focus on domestic violence victims, (2) the study did not include and discuss mental health interventions designed for domestic violence victims, (3) the study did not focus on COVID-19, and (4) the study was not published in English.

**Table 1:** Study inclusion criteria

Category	Criteria
Study population	Domestic violence victims
Language	English
Study focus	Mental health interventions for domestic violence victims amid COVID-19
Intervention	Conventional or technology-based interventions
Study outcome	Reporting of the design or the effect of the intervention

## Results

A total of 41 articles were included in the review. Both domestic violence and mental health issues are complex and multifaceted concepts [58]. To identify solutions to domestic violence victims’ mental health challenges amid COVID-19, we analyzed available literature and identified all potential direct and modifiable causes for domestic violence mental health issues. Overall, ‘direct’ reasons are more immediate and can be addressed cost-effectively with interventions. In contrast, ‘modifiable’ causes can be approached in both a timely and cost-effective manner. In other words, structural and systemic factors of domestic violence (e.g., imbalance of power between men and women) [59], whether they are salient before or during the pandemic, are beyond the scope of the current study.

**Fig. 1:**
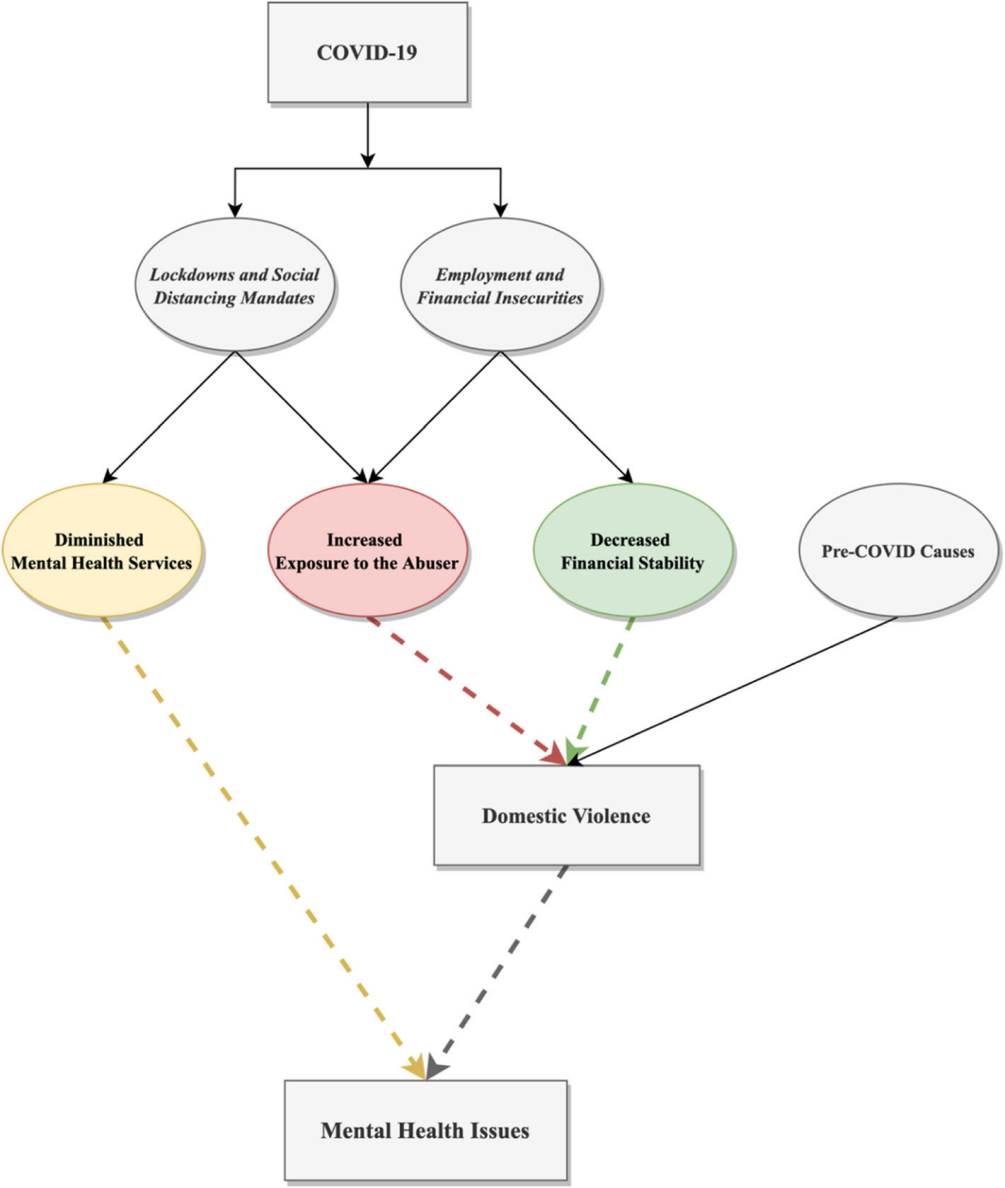
A schematic representation of factors that shape domestic violence victims’ mental health issues (dotted lines represent relationships discussed in the current study)

Our results showed that amid the COVID-19 pandemic, direct and modifiable factors that shaped women’s domestic violence, induced mental health issues, and exacerbate violence were: (1) increased exposure to the abuser, (2) decreased financial securities, and (3) diminished mental health services. Figure 1 displays illustrations of these factors and their interaction with domestic violence and mental health issues women face. Using these approaches as the framework, in the following section, we discussed specific solutions that can mitigate domestic violence victims’ mental health challenges amid COVID-19.

## Discussion

In this paper, we reviewed the literature and identified existing interventions that be adapted and applied to the COVID-19 context in order to safeguard and improve domestic violence victims’ mental health and well-being. To our knowledge, this is one of the first studies that examined this research topic. Overall, we identified three multidisciplinary approaches that have the potential to mitigate the unique mental health challenges domestic violence victims face amid COVID-19: (1) reduce exposure to the abuser, (2) eradicate financial insecurities, and (3) increase mental health services. In the context of this study, multidisciplinary approaches are defined as domestic violence strategies and solutions that require talents and resources from a diverse background, including but not limited to healthcare professionals, government officials, law enforcement, legal specialists, researchers, social workers, and volunteers. Considering the infrastructure and time required to improve domestic violence victims’ financial abilities, the following discussion will focus on solutions that center on reducing victims’ exposure to the abuser and increasing their access to mental health services. Detailed information on example nations’ domestic violence prevalence and interventions could be found in Table 2.

**Table 2:** Example nations’ domestic violence prevalence and interventions

	Australia	U.K.	China
Definition	“A set of violent behaviours between current or former intimate partners, where one partner aims to exert power and control over the other through fear. Domestic violence can include physical violence, sexual violence, emotional abuse and psychological abuse” [60].	“Any incident or pattern of incidents of controlling, coercive or threatening behaviour, violence or abuse between those aged 16 or over who are or have been intimate partners or family members1 regardless of gender or sexuality” [61].	An “infliction of physical, psychological or other harms among family members through means such as beating, restraints, maiming, restriction to physical liberty, as well as verbal abuse or intimidation” [62, 63].
Prevalence	In 2016, 1 in 6 women 15 years and older have experienced physical and/or sexual violence by a partner, 1 in 4 experienced emotional abuse, and 1 in 5 women have been sexually assaulted [64].	Available data show that, compared to the previous year, there is a 9% increase in domestic abuse-related crimes between March 2019 and March 2020 in England and Wales, the total number of which 758, 941 [65].	Academic studies suggest that domestic violence in china ranges from 10.2 to 65% [66–68].
Impact of COVID-19	Amid COVID-19 shelter-at-home mandates, there is a 5% hike in domestic abuse police call-outs and a 75% increase in online searches on domestic violence support [69]. Approximately 50% of women who experienced domestic violence prior to the pandemic reported more frequent or severe abuses during the pandemic [70].	In the U.K., during the 2020 Christmas season, police in West Midlands responded to 1250 domestic violence and abuse reports, a 60% increase compared to the same period in 2019 [71].	During the initial lockdown, domestic violence calls received by a nonprofit organization located in Hubei province tripled in February 2020 compared to the previous year [72].
Interventions	Health centers, counseling services, and call centers, such as the Safe Steps Family Violence Response Centre [73].	National and local sexual violence and domestic abuse services, organizations, and independent advisers, such as the Refuge’s National Domestic Abuse Helpline [74].	Non-legal interventions (e.g., shelters and hotlines) are lacking in terms of availability, accessibility, and awareness [68, 75].
Legislation Adequacy^a^	Wholly adequate	Wholly adequate	Some deficiencies

^a^*Legislation adequacy is measured by three concepts, namely, “the comprehensiveness of the meaning of domestic violence in the law”, “the appropriateness of the evidence required to prove domestic violence”, and “the acceptability of the legal punishments for domestic violence and protection of the victim”* [76]

### Reduce women’s exposure to the abuser

#### Face-to-face help-seeking solutions

It is important to note that even amid the intensive lockdowns, essential businesses, such as supermarkets and pharmacies, often remained available to sustain people’s basic physiological needs [77]. These societal behaviour changes present a valuable opportunity for health organizations and government agencies to establish help mechanisms to assist individuals in leaving their abusive environments amid and beyond the pandemic [78–80]. Disguising help-seeking activities, protecting their immediate safety, supermarkets or pharmacy staff have adopted coded messaging systems to sound the alarm [78].

Several European countries, such as France, Germany, Italy, Norway, the Netherlands, and Spain, have all adopted a coding messaging system, using a specifically designated code, ‘Mask 19’, for abused women to initiate help-seeking activities [78–80]. The U.K. has a similar codename scheme that employs the word “ANI” (stands for “Action Needed”) for domestic abuse victims to seek immediate help at participating pharmacies [81]. The way the system works is that by communicating the code word (either spoken or written), women can ask for help without attracting attention, even if the abuser is in close proximity [78–81]. The staff of the participating business can then contact police or social services on the woman’s behalf.

In countries without these systems, networks of pharmacies and chains of supermarkets could adopt coded systems to support domestic violence victims. Other groups of well-developed candidates are international food restaurant chains, such as McDonald’s, Pizza Hut, or Starbucks, where managers and staff often receive high-level training based on international standards [82–84]. Restaurant chains hold the advantage of time, in a sense that both domestic violence victims and their abusers are in a known location and confined space for a specific duration (i.e., dine-in time), allowing for police or social workers to respond and offer support. Considering that numerous food restaurant chains operate in China often have an international presence, utilizing these chain stores as potential points of support for domestic violence victims can benefit domestic violence victims living outside China.

#### Virtual ad-hoc help-seeking solutions

Fortunately, many countries have a telephone or virtual help-seeking service [79, 85, 86]. Virtual help-seeking has many advantages, ranging from transcending physical constraints, accessibility, cost-effectiveness, and can ensure anonymity, privacy, and security [85–87]. Online-based services also have the added advantage of being ‘silent services’, where individuals can share their stories and seek help without worrying about being eavesdropped on by their abusers [87]. Within the U.K., interaction with the domestic violence charity, ‘Respect’, soared 97% for calls, 185% for emails, and 581% for website visits during the first 3 weeks of the pandemic [56].

One of the most empowering online services formats is the ad-hoc online help-seeking platform, more commonly seen in high-income countries, including Norway, Germany, France, Spain, Italy, and Argentina [88]. Ad-hoc platforms provide a unique way to connect domestic violence victims with a vetted and comprehensive range of resources without having to search for another website to address their health and safety concerns or considerations [88]. The importance of having an ad-hoc online help-seeking platform available to domestic violence victims centers on the fact that having an all-encompassing system minimizes the time and effort needed for abused individuals to find online help. This access substantially reduces the barrier that could hinder help-seeking activities [89] and lower the chance these help-seeking activities might be interrupted by their abusers [87].

Some countries, such as China, have yet to have a national telephone or an ad-hoc online system that offers timely help to domestic violence victims [2]. Undoubtedly, there is a pronounced need for health organizations and government agencies to collaborate to develop an ad-hoc online help-seeking system to connect abused women in China with timely and comprehensive resources [5, 12, 90]. When designing and developing online help systems for domestic violence victims, access to information and resources in different formats is required. These formats may range from ones requiring high-speed bandwidth or access to those that can be accessed via low-tech devices [80]. When using these devices to access domestic violence resources, women will need to be careful to clear their browsing history or use a device unknown and inaccessible to the abuser.

### Increase mental health resources

#### Shelter homes amid COVID-19

A well-established system that addresses the physiological and safety needs of individuals experiencing domestic violence, ranging from food, shelter, safety to access to medicine [91], is essential. Countries like Canada have made sure domestic violence shelters remain open amid the pandemic; other countries, such as France, initiated emergency shelter provisions that allow the government to repurpose hotels as safe houses [79]. For countries without adequate infrastructure or resources before the onset of the pandemic [2], the French example may have greater relevance. In other words, health organizations and government agencies in China, for example, could consider how to repurpose hotels or housing facilitates, especially those vacant amid COVID-19, as shelter homes for individuals in times of urgent needs.

Considering international and domestic tourism has suffered considerably from COVID-19’s adverse impacts [92, 93], utilizing hotels amid the pandemic could be a cost-effective approach for society at large to organize a collaborative response to domestic violence. Furthermore, the government can also explore whether repurposing hotels as shelter homes is a possible a long-term plan, as it is common for hotels to have vacant rooms. In collaboration with local support services, a potential scheme could see some form of compensation for the use of these facilities. This suggestion will require extensive hotel staff training, possibly carried out with local and national support services, that will support staff in managing challenging situations that may arise, such as a spouse or partner seeking the whereabouts of an individual experiencing domestic violence.

#### Education programs

It is important to note that domestic violence can result from failing social systems, and the issue often takes time and support from others to address [3]. However, what is also important to acknowledge is the indispensable role of taking the initiative to seek help [94–96]. One cost-effective way of increasing seeking behaviour is through integrated campaign interventions. In the context of this study, integrated campaign interventions are the use of communication and marketing resources to deliver persuasive messages to domestic violence victims, aiming to elicit positive changes in attitudes and behaviors towards help-seeking behaviours.

Evidence shows that several countries and non-governmental organizations (NGOs) have initiated public health campaigns to address domestic violence prevalence and severity [80, 97–99]. Most of these campaigns, including those sponsored by individual countries such as the Netherlands and Kosovo, focus on informing abused women the importance of seeking domestic violence services and raise awareness in the society at large about the prevalence and the need to “Report Violence, Save Lives” [80, 97–99]. In addition to these two types of campaigns, governments may consider extending their campaign's scope to the abusers. Informing domestic violence offenders of available laws, regulations, and the social consequences associated with domestic violence abuse, oftentimes will help minimize their likelihood to engage in abuse or in further abuse.

A growing body of research has examined intervention programs for specific gender abusers in the domestic violence context [100–102]. These interventions are categorized into broad dyad-centered programs that often include other members of the abusers’ community. Research indicates that dyad-based programs typically include more comprehensive domestic violence care community members as well (e.g., health experts, social workers, etc.), and have great potential in generating desirable intervention outcomes [102, 103]. Combining occurrence, prevalence, and severity, domestic violence is influenced by a connected and comprehensive range of factors; a practical solution could involve the design of campaigns that educate and inform the abusers of the consequences. Educating community members to intervene and help to protect women from further damages of domestic violence is vital.

#### Escape plans

There is a need for more training on creating an escape plan if an individual chooses to leave their abuser(s) during a lockdown. As more women are working from home, staying home to watch children, or in lockdown due to the pandemic, it may make leaving an abuser nearly impossible [8–10], especially if the abusers are also home or working from home more often. It may not be possible for the victim to leave if the abuser is still present in the home and, therefore, well-developed escape plans could safely support victims of abuse [104, 105]. Escape from an abuser can be dangerous, and both the benefits and risks will need to be considered for the particular situation; especially if children are involved, as “strategies that don’t match risks and circumstances may not improve safety and may even increase risks” [104].

One strategy is to have organizations and businesses make resources, tips, and supplies available to pick up discreetly and can be used to plan for an escape or to help enable women to make up an escape bag. Emergency escape plans may include advice or information surrounding access to extra money or opening a new bank account in their name, attaining sets of keys, clothes, a prepaid phone to avoid tracking, or copies of essential documents or necessities for children [104, 105]. They may also consider alternative transportation, as an abuser could track a family vehicle [104, 105]. A designated place to go, whether a shelter, a newly rented apartment, a hotel, a friend, or a family member’s house at a location unknown to the abuser, should be considered as part of their escape plan [104, 105].

The lack of a detailed plan places the victim and their children at an unforeseen risk of harm. Having a plan, and perhaps a backup plan should an abuser identify the situation, could be adjusted should there be a postponement until leaving is safe [104, 105]. Information regarding how to dispose of smart devices, phones, or watches is vital; these technologies may pair to a household computer and have the potential to be used as a tracking device. In western countries, such as the U.S., often have specially designed and designated protection programs for abuse victims [106]. Health organizations and government agencies could use these existing examples to establish tailored programs to support domestic violence victims’ needs.

#### Laws and regulations

One approach that can address the prevalence of domestic violence in countries is a national domestic violence registration system. This concept is similar to the National Sex Offender Registry seen in western countries, such as the United States, where a searchable public record is available for concerned parents and educators to use as a reference to stave off potential dangers of these abusers to children [107]. Having a national domestic violence registration system has the potential to allow individuals to check whether their potential partners have a violent and abusive history before forging a relationship with perpetrators.

In the context of applying this approach, this avant-garde concept has already been implemented in one Chinese city, Yiwu [108]. The searchable database enacted by the Yiwu government allows people to search domestic abuse offenders from across the country, including those convicted, issued with a restraining order, or sentenced to detention for domestic abuse since 2017 [108]. Several documents are needed to inquire or initiate this process: (1) the I.D. of both individuals, (2) their official marriage application, and (3) signed a confidentiality agreement.

While this database has been available since July, 2020, data on how this system has helped avoid instances of domestic violence has yet to become available. What is clear, though, is that individuals in Yiwu will have access to critical insights into their fiancée before they further develop their relationships with them. The Yiwu model will also provide invaluable insights that other municipal governments and the central government can borrow to build their systems to prevent and control domestic violence. It is important to emphasize that the government should offer assistance in filing for search applications among people who may have difficulties filing the applications independently, such as the visually impaired or individuals who may be illiterate, so unnecessary barriers can be reduced.

#### Technology-based mental health solutions

Until COVID-19 vaccinated becomes a universal fact [109–111], lockdowns and social distancing mandates will likely continue to be a solution when there are future outbreaks or infections. This barrier to physical contact paves the way for technology-based interventions to address domestic violence victims’ mental health issues [112, 113]. Technology-based interventions could, in this context, act as a means to manage or support the health promotion strategies to produce “accessible and affordable health solutions to the target audience” [114].

Accumulating evidence indicates that technology-based interventions could generate a myriad of beneficial effects on victims in violence against women to reduce their symptoms of depression, anxiety, and exposure to intimate partner violence [115–117]. Findings also indicate that technology-based interventions can often result in better outcomes compared to conventional approaches (e.g., in-person interventions) [118]. Compared to traditional intervention approaches, research also suggests that women are more likely to share their experiences in technology-based interventions [119]. This finding is extremely relevant, as due to the imbalance of power between the abuser and the abused [120], individuals are often reluctant to engage in help-seeking activities that are crucial in separating them from their abusive environments.

Technology-based interventions have unique advantages that make them an ideal intervention delivery option in the context of COVID-19: (1) cost-effectively tailored to the unique needs and preferences of the target audience, (2) adopted to deliver health solutions remotely via transcending physical barriers such as time and money needed for transportation, (3) can be developed, delivered, and accessed cost-effectively, and (4) can be engineered to protect the target audience’s privacy and emotional concerns as they facilitate user anonymity in the health solution delivery process [121–123].

Another reason why technology-based mental health services are essential is that some countries witness growing waiting lists and a disproportionate number of support workers. For example, a significant shortage of psychiatrists or other mental healthcare professionals in China means a delay in face-to-face consultations [124–126]. This lack of mental health professionals suggests that health organizations and government agencies need to integrate international help into their online mental health services, such as help gained from countries that endorse the Istanbul Convention, a slightly flawed yet urgently needed legal framework that aims to protect women from violence at a pan-European level [127–130]. With advances in technology and globalization, multilingual mental health experts worldwide are all potential sources of help for domestic violence victims. Similar to the concept of Doctors without Borders [131], health organizations and government agencies should consider establishing a system that can connect all available international mental health experts across the world with individuals experiencing or at risk of domestic violence [132].

#### Limitations

While this study fills important research gaps, it is not without limitations. First, this study is not a systematic review. We did not follow the Preferred Reporting Items for Systematic Reviews and Meta-Analyses (PRISMA) procedure [133] in our review process. This, in turn, suggests that our study is limited in its reproducibility and replicability. However, it is important to note that due to a lack of available interventions developed amid COVID-19, a systematic review of mental health interventions for domestic violence victims tailored to the COVID-19 context may not be practical at the time of the study.

## Conclusions

Domestic violence is a public health crisis. It affects both women and men, and it could interrupt generations of people's aims, aspirations, or ambitions, undermine their chance at a violence-free life. COVID-19 both amplified and introduced mental health challenges domestic violence victims face, many thanks to the human and economic consequences the pandemic inflamed. However, there is a lack of mental health interventions tailored to domestic violence victims in the COVID-19 context. In this study, we identified practical and multidisciplinary interventions that can be effectively adopted and applied to address the mental health challenges domestic violence victims face amid, and possibly beyond, the current pandemic. Future research could explore additional practical solutions to alleviate the many health burdens domestic violence victims shoulder. Overall, domestic violence victims need more resources and help to no longer suffer in silence, in pain, alone, or afraid during the current and future pandemics.

## Data Availability

Data available upon reasonable request.
